# Computed Tomography Angiography in Pediatric Pulmonary Hypertension: Evaluating MPA-to-Aortic Ratios as Diagnostic Markers

**DOI:** 10.3390/diagnostics15131614

**Published:** 2025-06-25

**Authors:** Ali Nazım Güzelbağ, Serap Baş, Demet Kangel, Muhammet Hamza Halil Toprak, Ahmet Saki Oğuz, Selin Sağlam, İbrahim Cansaran Tanıdır, Erkut Özturk

**Affiliations:** 1 Department of Pediatric Cardiology, Saglik Bilimleri University, Basaksehir Cam and Sakura City Hospital, 34480 Istanbul, Turkey; demetkangel@gmail.com (D.K.); muhammedhamzatoprak@hotmail.com (M.H.H.T.); ahmetsaki@gmail.com (A.S.O.); cansaran@yahoo.com (İ.C.T.); erkut_ozturk@yahoo.com (E.Ö.); 2 Department of Radiology, Saglik Bilimleri University, Basaksehir Cam and Sakura City Hospital, 34480 Istanbul, Turkey; serapbas579@gmail.com; 3 Department of Anesthesiology, Saglik Bilimleri University, Basaksehir Cam and Sakura City Hospital, 34480 Istanbul, Turkey; selinsaglams@gmail.com

**Keywords:** pulmonary hypertension (PHT), computed tomography angiography (CTA)

## Abstract

**Background:** Pulmonary hypertension (PHT) is a rare but serious condition in children, requiring early diagnosis to prevent right ventricular failure. Non-invasive imaging modalities such as computed tomography angiography (CTA) have gained importance in assessing vascular changes, including main pulmonary artery (MPA) dilatation, increased vessel stiffness, and elevated pulmonary vascular resistance, which are characteristic of pulmonary hypertension (PHT). **Objective:** This study aimed to evaluate the diagnostic value of the main pulmonary artery-to-ascending aorta (MPA/AA) and main pulmonary artery-to-descending aorta (MPA/DA) ratios on CTA in pediatric patients with confirmed PHT. **Methods:** In this retrospective cohort study, 76 pediatric patients who underwent both cardiac catheterization and thoracic CTA were included. Patients were divided into PHT (mean pulmonary artery pressure ≥ 25 mmHg) and non-PHT groups. Vascular measurements were obtained from CTA, and MPA/AA and MPA/DA ratios were calculated. Statistical analyses included Mann–Whitney U tests and ROC curve analysis. **Results:** The MPA diameter and MPA/AA and MPA/DA ratios were significantly higher in the PHT group compared to controls (*p* < 0.05). ROC analysis showed strong diagnostic performance for both ratios. The MPA/DA ratio had an AUC of 0.927 with 78.5% sensitivity and 94% specificity at a cut-off value of 1.85. The MPA/AA ratio had an AUC of 0.896 with 76.5% sensitivity and 95% specificity at a cut-off value of 1.25. **Conclusions:** Both MPA/AA and MPA/DA ratios are reliable non-invasive indicators of pediatric PHT, with the MPA/DA ratio demonstrating slightly higher diagnostic accuracy. These findings support the use of CTA-derived vascular ratios, especially MPA/DA, as effective screening tools in clinical practice.

## 1. Introduction

Pulmonary hypertension (PHT), although rare in children, is a potentially life-threatening condition that can lead to significant morbidity and mortality [1]. Therefore, early diagnosis and appropriate treatment are critical to preserve right ventricular function and improve survival in pediatric patients [2]. PHT is defined as a mean pulmonary artery pressure higher than 25 mmHg at rest [3]. Pathophysiologically, PHT is associated with increased resistance in the pulmonary vascular bed due to mechanisms such as endothelial dysfunction, vascular smooth muscle hyperplasia, intimal fibrosis, and microthrombosis [4]. These changes progressively increase right ventricular pressure overload, ultimately leading to right heart failure [5].

Clinical manifestations of PHT are generally nonspecific, and patients often present with symptoms of dyspnea, exercise intolerance, fatigue, syncope, and chest pain. As the disease progresses, signs of right heart failure and peripheral edema may develop [6]. While transthoracic echocardiography is typically the first-line imaging modality in the diagnostic process, definitive diagnosis is made by right heart catheterization [7]. However, due to its invasive nature and associated risk of complications, non-invasive imaging techniques have increased in importance in recent years [8].

With advances in imaging technology, computed tomography angiography (CTA) has become increasingly used in the diagnosis and follow-up of both congenital heart diseases and pulmonary diseases due to its lower radiation exposure and better image quality [9]. In children with suspected PHT or other cardiopulmonary symptoms, CT provides a valuable non-invasive alternative for evaluation [10]. CT angiography provides an indirect assessment of PHT by measuring the diameter of the main pulmonary artery (MPA) and ratios such as that of the MPA to the ascending aorta (AA) or the descending aorta (DA) [11]. In pediatric populations, absolute vascular diameters are influenced by factors such as age, sex, and body surface area, which may limit their reliability. Therefore, ratio-based measurements like MPA/AA and MPA/DA provide a more standardized and age-independent method for assessing vascular abnormalities.

Studies have shown an association between MPA dilatation and PHT in adults, with MPA diameters > 29 mm and MPA/AA ratios > 1:1 considered predictive [12]. Similar findings have been reported in pediatric populations. However, in children, vessel diameters are influenced by variables such as age, sex, and body surface area, making ratio-based evaluations more reliable than absolute measurements [13].

As reported by Caro-Domínguez et al., the average MPA/AA ratio in healthy children is approximately 1.09, and a cut-off value of 1.3 may serve as a predictive value for PHT [14]. On the other hand, pathologies involving the aortic valve or aortic arch may change the diameter of the ascending aorta, which may affect the accuracy of this measurement. For this reason, several researchers have proposed the MPA/DA ratio as a more reliable diagnostic marker. One study reported that an MPA/DA ratio greater than 1.8 was a strong predictor of PHT, with a specificity of 91% and a positive predictive value of 88%.

In our study, we evaluated pediatric patients with confirmed PHT based on right heart catheterization and without additional aortic or arch anomalies. We compared patients with a mean pulmonary artery pressure above 25 mm Hg with those with lower pressures, analyzing the ratio of the MPA to both the ascending and descending aorta.

## 2. Materials

Our study was a retrospective case–control study. The study was designed in accordance with the tenets of the Declaration of Helsinki and obtained the necessary approval from the local ethics committee. All patients were evaluated as part of routine clinical care, and no additional imaging or intervention was performed beyond standard practice. The data presented in this study were collected retrospectively from medical records under the approval of the local ethics committee (protocol code: 2025/118; date: 14 May 2025).

Patients between 0 and 18 years of age who underwent catheter angiography between January 2022 and January 2025 and who had computed tomographic angiography of the heart within 3 months before or after catheter angiography were included in the study. Patients were considered to have pulmonary hypertension if their mean pulmonary artery pressure measured by cardiac catheterization was ≥25 mmHg. The control group consisted of patients with a mean pulmonary artery pressure < 25 mmHg as measured by cardiac catheterization. Patients with additional cardiac anomalies that could potentially cause pulmonary hypertension, such as hypoplastic ventricles, atrioventricular (AV) valve anomalies, AV valve regurgitation, aortic valve anomalies, aortic valve stenosis or regurgitation, and aortic arch hypoplasia, were excluded from the study.

Pulmonary vascular evaluations were conducted using cardiac catheterization performed under general anesthesia via femoral access, employing the Philips^®^ Artis bi-plane AZURION 7 B12 system (Philips Medical Systems, Eindhoven, The Netherlands). When sedation was necessary, intravenous administration of midazolam and/or fentanyl was provided by a cardiac anesthesiologist. To ensure patient safety, adequate hydration was maintained before and after the intervention, and renal function was assessed pre- and post-procedure. Pressure measurements and oxygen saturation levels were recorded in various cardiac chambers, including the aorta, pulmonary artery, and vena cava, utilizing fluid-filled catheter systems. Angiocardiographic imaging was performed with an isoosmolar, non-ionic contrast agent (iodixanol) at a maximum dose of 4 mL/kg. Right ventricular angiograms were acquired in both the anteroposterior projection with cranial tilt and the left anterior oblique view to assess the right ventricular outflow tract, pulmonary artery morphology, and ventricular contractility. Conventional catheter angiography (CCA) was conducted by a pediatric cardiologist possessing over a decade of expertise in diagnosing congenital heart anomalies. The angiographic data obtained during cardiac catheterization were independently interpreted by a cardiologist blinded to the findings of computed tomography angiography (CTA) and echocardiography (ECHO).

Cardiac CT imaging was conducted using a 640-slice single-source CT scanner (Aquilion ONE, GENESIS Edition; Canon Medical Systems, Otawara, Tochigi, Japan) featuring a wide 16 cm detector and employing the Adaptive Iterative Dose Reduction 3D Enhanced (AIDR 3D Enhanced) algorithm. A prospective ECG-gated approach was applied during a single cardiac cycle for all subjects. Images were obtained in Volume Axial mode (rotation time: 0.35 s; scan length: 80–160 mm), with tube current managed via automatic exposure modulation [15]. Low kilovoltage settings (80–100 kV) were selected to optimize iodine contrast, with 80 kV used for patients under 35 kg and 100 kV for those over. Each patient received an intravenous bolus of iodinated contrast medium (Kopaq 300 mgI/mL; Onko&Kocsel Pharmaceuticals, Kocaeli, Turkey) at 1.5 mL/kg, followed by a 10–20 mL saline flush using a dual-head power injector (MEDRAD, Bayer HealthCare, Beek, The Netherlands). The injection rate ranged from 0.7 to 3.5 mL/kg, adjusted according to catheter caliber and patient size. Undiluted contrast was used throughout. Scans were performed without breath-holding or sedation, and an experienced cardiac radiologist supervised all examinations. Imaging acquisition targeted the initial contrast passage through the cardiovascular structures, centering the acquisition window at 45% of the R–R interval in patients with heart rates exceeding 90 bpm [16]. Scanning was paused during phases deemed non-essential. For each individual, the most motion-free cardiac phase closest to the predefined target was retrospectively selected by the radiologist. Data were reconstructed at a 0.5 mm slice thickness using a standard kernel and the AIDR 3D Enhanced algorithm.

Post-processing included multiplanar reconstruction (MPR), maximum intensity projection (MIP), and 3D volume rendering (VR). Radiation dose metrics—including dose-length product (DLP), computed tomography dose index volume (CTDIvol), and scanned anatomical area—were documented for every examination. The dose delivered to patients was evaluated in terms of CT dose index and dose-length product. Effective doses were derived from chest conversion factors using the following formula.

- The CTDI and DLP values referenced a 32 cm phantom, while the effective dose (ED) was calculated by multiplying the DLP by a factor of 2 to adapt to a 16 cm phantom model [17].
- Conversion coefficients specific to neonates and infants (0.039 mSv/(mGy·cm)) were applied based on age group for accurate dose estimation [18].
- ED (mSv = DLP (mGy.cm).(mSv.mGy^−1^.cm^−1^)). The CT dose index, dose-length product, and conversion factor were expressed for a 32 cm body phantom reference [19].

CTA was performed by a pediatric cardiovascular radiologist with at least 15 years of experience in congenital heart imaging and blinded to the reports of right heart catheterization. The diameters of the descending aorta (DA), ascending aorta (AA), and main pulmonary artery (MPA) in the short axis were measured at the level of the diaphragmatic outlet, ascending aorta, and pulmonary bifurcation, respectively. Only the luminal diameter was included, excluding the vessel wall. All measurements were performed in the axial plane, perpendicular to the long axis of each vessel, using mediastinal window settings (width: 400 HU; height: 40 HU). The diameters of the AA and MPA were assessed on axial slices with a thickness of 5 mm (2.5 mm for children younger than 2 years) and up to 100% of enlargement. Multiplanar reconstructions or area calculations were not used to improve reproducibility.

To maintain consistency and reduce interpretation bias among different imaging techniques, each modality was assessed independently. Radiologists responsible for interpreting computed tomography angiography (CTA) and cardiologists reviewing conventional catheter angiography (CCA) were blinded to findings from other imaging methods. Every imaging dataset was individually analyzed by specialists with substantial expertise in congenital heart disease diagnostics, adhering strictly to established standardized protocols.

### Statistical Analysis

Statistical analysis was performed to compare the vascular measurements between pediatric patients with and without pulmonary hypertension (PHT), as confirmed by right heart catheterization. Descriptive statistics, including medians, interquartile ranges (IQRs), means, and standard deviations, were calculated for all continuous variables to summarize their distributions within the study groups. To enhance diagnostic interpretation, several anatomical ratios were derived, including the main pulmonary artery-to-descending aorta (MPA/DA) ratio and the main pulmonary artery to ascending aorta (MPA/AA) ratio. These variables were calculated for each participant and used in subsequent analyses. Since the data did not follow a normal distribution, the non-parametric Mann–Whitney U test was applied to assess differences between the PHT and non-PHT groups. A two tailed *p*-value of less than 0.05 was considered statistically significant. In addition, receiver operating characteristic (ROC) curve analysis was conducted to evaluate the diagnostic performance of the MPA/DA and MPA/AA ratios in detecting PHT. The area under the curve (AUC), sensitivity, specificity, positive predictive value (PPV), negative predictive value (NPV), and positive likelihood ratio (LR^+^) were calculated for each ratio. ROC curves were visualized with matplotlib, and a reference line representing random classification was included. All statistical analyses were performed using SPSS version 25.0 (SPSS Inc., Chicago, IL, USA), and a *p*-value of <0.05 was considered statistically significant.

## 3. Results

A total of 76 pediatric patients were included in the study, with 41 patients with pulmonary hypertension (PHT group) and 35 patients without pulmonary hypertension (non-PHT group) that was confirmed by right heart catheterization. Baseline clinical characteristics of the PHT and non-PHT groups are shown in Table 1. There were no statistically significant differences between the groups in terms of gender, age, weight, weight percentile, weight SDS, height, height percentile, height SDS, body mass index (BMI), BMI percentile, BMI SDS, body surface area (BSA), peripheral oxygen saturation (SpO_2_), computed tomography dose index volume (CTDIvol), dose-length product (DLP), effective dose (ED), and contrast volume.

Pulmonary artery pressure was higher in the PHT group (35.5 [28.0–42.5] mmHg) compared to the non-PHT group (16 [13.75–19.0] mmHg); however, this difference was not statistically significant (*p* = 0.0508). Although the diameters of the ascending aorta (15.85 [12.8–17.62] vs. 14.25 [11,12,13,14,15,16,17,18,19] mm, *p* = 0.37) and descending aorta (10.5 [8.52–12.92] vs. 10.1 [8.2–12.69] mm, *p* = 0.46) were slightly greater in the PHT group, these differences were not statistically significant. In contrast, the main pulmonary artery (MPA) diameter was significantly bigger in the PHT group (19.8 [16.4–22.1] mm) than in the non-PHT group (16.2 [11.6–18.4] mm) (*p* = 0.04). Similarly, the MPA/DA ratio (2.3 [1.88–2.40] vs. 1.58 [1.36–1.67], *p* = 0.0027) and the MPA/AA ratio (1.38 [1.25–1.52] vs. 1.16 [0.86–1.50], *p* = 0.0116) were both significantly higher in the PHT group, suggesting their potential diagnostic value in identifying pulmonary hypertension in pediatric patients. The diameters of the main pulmonary artery (MPA), descending aorta (DA), and ascending aorta (AA), as well as the ratios of MPA to DA (MPA/DA) and MPA to AA (MPA/AA), are shown in Table 2.

Subgroup analysis across different pediatric age groups (0–2, 2–7, 7–12, and 12–18 years) showed that both MPA/DA and MPA/AA ratios were consistently higher in the PHT group compared to the non-PHT group. Statistically significant differences were observed in almost all age categories for both ratios, especially for MPA/DA, which showed robust discriminatory power in all subgroups. The only exception was the MPA/AA ratio in the 7–12-year group, where the difference did not reach statistical significance (*p* = 0.078). Overall, the results show that these vascular ratios, especially the MPA/DA ratio, are reliable diagnostic markers for PHT in all age groups. The age-based comparison of pulmonary artery-to-aorta ratios (MPA/AA and MPA/DA) in pediatric pulmonary hypertension is shown in Table 3.

**Figure 1:**
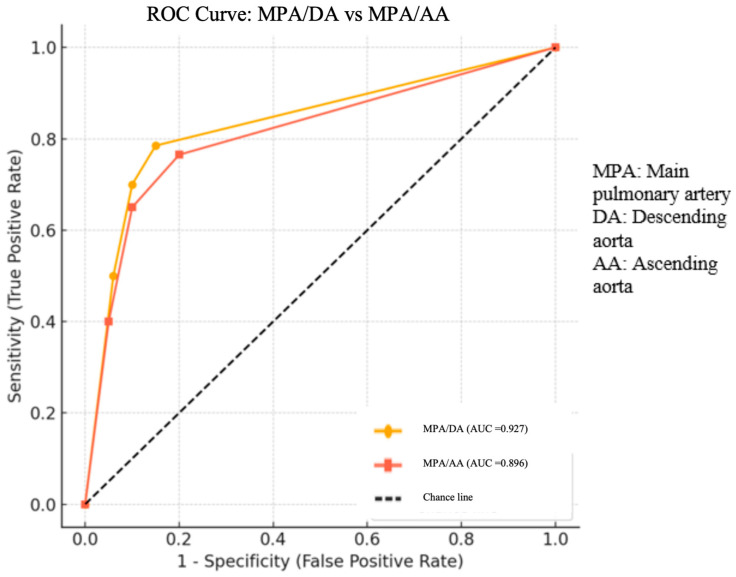
The ROC analysis comparing the diagnostic performance of the MPA/DA and MPA/AA ratios.

ROC analysis showed that a cut-off value of 1.85 for the MPA/DA ratio had a sensitivity of 78.5%, a specificity of 94%, a positive predictive value (PPV) of 91%, and a positive likelihood ratio (LR^+^) of 7.5, with an AUC of 0.927. For the MPA/AA ratio, the cut-off value of 1.25 also showed high specificity (95%) and PPV (89.7%), with a sensitivity of 76.5% and an AUC of 0.896. The ROC analysis comparing the diagnostic performance of the MPA/DA and MPA/AA ratios in identifying pediatric pulmonary hypertension is shown in Figure 1.

## 4. Discussion

The aim of this study was to evaluate the diagnostic accuracy of the main pulmonary artery (MPA)-to-ascending or descending aorta ratio on CT angiography in children with suspected pulmonary hypertension (PHT). In our study, we compared the MPA/DA and MPA/AA ratios between patients with and without PHT confirmed by catheter angiography and assessed by thoracic CT angiography. The most comprehensive evaluation of pulmonary artery dimensions in an adult population using computed tomography was conducted in the Framingham Heart Study, which included 3171 individuals with a mean age of 51 years, 51% of whom were male [20]. Among 706 asymptomatic participants without any cardiopulmonary risk factors, the mean main pulmonary artery (MPA) diameter was reported as 24.7 mm (SD: 2.7), and the mean MPA-to-ascending aorta (AA) diameter ratio was calculated as 0.80 (SD: 0.09). In the pediatric population, Akay et al. assessed the mean MPA diameter in a cohort of 133 children with no history of cardiac or pulmonary disease [21]. This study showed that pulmonary artery diameter can be affected by multiple factors and concluded that the ratio between the main pulmonary artery (MPA) and other thoracic vessels may serve as a more specific parameter for predicting pulmonary hypertension (PHT). In addition, a study of 50 patients carried out by C. S. Ng et al. found that the MPA-to-ascending aorta ratio was significantly increased in patients with a mean pulmonary artery pressure of 20 mmHg or higher [22].

In a meta-analysis by Yongchun Shen et al., both main pulmonary artery diameter and the pulmonary artery-to-aorta (PA:A) ratio were shown to be useful diagnostic indicators of pulmonary hypertension, supporting their use as non-invasive imaging tools in clinical practice [23].

In adults, a main pulmonary artery (MPA)-to-ascending aorta (AA) ratio greater than 1 is considered predictive of pulmonary hypertension (PHT), with a reported positive predictive value (PPV) of 96% and a specificity of 92%. However, a study by Compton GL et al. showed that the MPA/AA ratio in individuals without risk factors for PHT was 1.085, which is in contrast to the adult population [24]. In the continuation of the same study, this ratio was found to be 1.1, and the authors attributed this finding to the younger age of the patient population. Similarly, in our study, the MPA/AA ratio was found to be 1.16 in the control group, which consisted of children with no pulmonary hypertension confirmed by catheter angiography. The relatively higher value observed in our cohort may also have been due to the younger age of the study population. Our findings are consistent with those of Compton et al., who emphasized that the MPA-to-ascending aorta (AA) ratio in the pediatric population is typically greater than 1.0, in contrast to cut-off values based on adults. This finding highlights the importance of establishing age-specific reference values when interpreting CT-based vascular measurements in children being evaluated for pulmonary hypertension.

In the study by Muankwan Saetung et al., the MPA/AA and MPA/DA ratios were found to be significantly higher in 45 children diagnosed with pulmonary hypertension (PHT) compared with an age-, sex-, and body surface area (BSA)-matched control group without PHT risk factors. In the control group, the mean MPA/AA ratio was reported to be 1.1, and a cut-off value of 1.3 was proposed for the diagnosis of PHT, resulting in a positive predictive value (PPV) of 85.7% and a specificity of 93% [25]. These results were consistent with those reported by Pablo Caro-Domínguez et al., in whose study the mean MPA/AA ratio in healthy children was 1.11 and a cut-off of 1.3 achieved a PPV of 97%. However, in the study by Muankwan Saetung et al., the discriminative power of the MPA/AA ratio was lower, with an area under the receiver operating characteristic (ROC) curve (AUC) of 0.667, compared with the AUC of 0.94 reported by Caro-Domínguez et al. [14,25].

In our study, there was no statistically significant difference in the diameters of the ascending and descending aortas between the two groups. However, the diameter of the main pulmonary artery (MPA) was found to be significantly larger in the PHT group. Similarly, the MPA/DA and MPA/AA ratios were significantly higher in patients with pulmonary hypertension. Consistent with our findings, Pablo Caro-Domínguez et al. also reported a higher MPA/AA ratio in the PHT group in their study [14]. Similarly, Muankwan Saetung et al. found that both the main pulmonary artery diameter and MPA/DA and MPA/AA ratios were significantly higher in children with pulmonary hypertension [25].

In our study, ROC analysis showed that a cut-off value of 1.85 for the MPA/DA ratio had a sensitivity of 78.5%, a specificity of 94%, a positive predictive value (PPV) of 91%, and a positive likelihood ratio (LR^+^) of 7.5, with an AUC of 0.927. These results indicate that the MPA/DA ratio has high discriminatory power for the diagnosis of pediatric pulmonary hypertension (PHT). For the MPA/AA ratio, the cut-off value of 1.25 also showed high specificity (95%) and PPV (89.7%), with a sensitivity of 76.5% and an AUC of 0.896. Although both ratios demonstrated strong diagnostic performance, the MPA/DA ratio showed slightly superior overall accuracy based on ROC curve analysis. These findings support the use of the MPA/DA ratio as a more sensitive and reliable radiological marker for the diagnosis of pediatric PHT, suggesting that it may be preferred in clinical practice.

Although the difference in mean pulmonary artery pressure between the groups did not reach statistical significance (*p* = 0.0508), this finding may be attributed to the composition of the control group, which consisted of patients referred for catheterization due to clinical suspicion of pulmonary hypertension but who were subsequently confirmed to have normal hemodynamic parameters. Several of these individuals likely presented with conditions that can transiently elevate pulmonary pressures or mimic early-stage PHT, including small left-to-right shunts, self-limited neonatal pulmonary pressure elevations, and chronic pulmonary conditions of non-cardiac origin.

## 5. Conclusions

This study showed that the main pulmonary artery-to-ascending aorta (MPA/AA) and main pulmonary artery-to-descending aorta (MPA/DA) ratios, measured by computed tomography angiography (CTA), serve as reliable non-invasive indicators for the diagnosis of pediatric pulmonary hypertension (PHT). Both ratios were significantly elevated in patients with confirmed PHT compared to controls, with the MPA/DA ratio having better diagnostic performance based on receiver operating characteristic (ROC) analysis. Given its higher sensitivity, specificity, and positive predictive value, the MPA/DA ratio appears to be a more robust and consistent radiologic marker for identifying PHT in children. These findings support the routine incorporation of MPA/DA ratio assessment into clinical practice, as its high diagnostic accuracy makes it particularly useful for the early screening of pediatric patients at risk of pulmonary hypertension, enabling timely diagnosis and intervention.

## Figures and Tables

**Table 1:** Demographic and clinical characteristics of pediatric patients with and without pulmonary hypertension.

			*p*-Value
Sex (male)	Non-PHT group	%54 male (*n* = 19/35)	0.672
	PHT group	%66 male (*n* = 27/41)	
Age (month)	Non-PHT group	42.1 (15.2–136.4)	0.789
	PHT group	49.3 (14.9–152.1)	
Weight (kg)	Non-PHT group	14.3 (11.2–18.3)	0.543
	PHT group	16.4 (12.3–19.1)	
Weight (percentile)	Non-PHT group	29.12 (20.8–44.6)	0.234
	PHT group	35.2 (21.4–52.4)	
Weight (SDS)	Non-PHT group	−0.49 (−1.11–0.32)	0.098
	PHT group	−0.38 (−0.89–0.21)	
Height (cm)	Non-PHT group	97.8 (80.2–112.4)	0.275
	PHT group	105.2 (84.3–115.6)	
Height (percentile)	Non-PHT group	41.2 (26.4–50.8)	0.765
	PHT group	43.2 (24.2–48.9)	
Height (SDS)	Non-PHT group	−0.19 (−1.12–0.87)	0.389
	PHT group	−0.12 (−0.96–0.79)	
BMI kg/m^2^	Non-PHT group	14.95 (11.82–17.4)	0.654
	PHT group	14.82 (10.98–16.5)	
BMI (percentile)	Non-PHT group	33.36 (20.8–45.4)	0.432
	PHT group	27.82 (22.5–42.8)	
BMI (SDS)	Non-PHT group	−0.43 (−1.32–0.08)	0.685
	PHT group	−0.51 (−1.25–0.32)	
Saturation (spo2)	Non-PHT group	99.4 (98.2–99.1)	0.145
	PHT group	98.8 (98.7–99.3)	
CTDIvol (mGy)	Non-PHT group	9.08 (7.65–16.78)	0.346
	PHT group	9.96 (8.12–15.65)	
DLP (mGycm)	Non-PHT group	35.18 (28.65–48.32)	0.295
	PHT group	43.92 (31.28–52.4)	
ED (mSv)	Non-PHT group	1.98 (1.62–2.32)	0.543
	PHT group	2.01 (1.88–2.68)	
Contrast (cc)	Non-PHT group	15.65 (12.1–25.3)	0.278
	PHT group	14.82 (11.9–28.2)	

ED: Effective Dose, DLP: Dose-Length Product, CTDIvol: Computed Tomography Dose Index Volume, BMI: Body Mass Index, PHT: Pulmonary Hypertension, Non-PHT: Without Pulmonary Hypertension.

**Table 2:** The diameters of the main pulmonary artery (MPA), descending aorta (DA), and ascending aorta (AA) and the ratios of MPA to DA (MPA/DA) and MPA to AA (MPA/AA) in children with and without pulmonary hypertension (PHT).

	Non-PHT (*n* = 35)	PHT (*n* = 41)	*p*-Value
Pulmonary artery pressure	16 (13.75–19.0)	35.5 (28.0–42.5)	0.0508
Ascending aorta (mm)	14.25 (11–19)	15.85 (12.8–17.62)	0.37
Descending aorta (mm)	10.1 (8.2–12.69)	10.5 (8.52–12.92)	0.46
Main pulmonary artery (mm)	16.2 (11.6–18.4)	19.8 (16.4–22.1)	0.04
MPA/DA ratio	1.58 (1.36–1.67)	2.3 (1.88–2.40)	0.0027
MPA/AA ratio	1.16 (0.86–1.50)	1.38 (1.25–1.52)	0.0116

MPA: main pulmonary artery, DA: descending aorta, AA: ascending aorta, PHT: pulmonary hypertension, Non-PHT: without pulmonary hypertension.

**Table 3:** Age-based comparison of pulmonary artery-to-aorta ratios in pediatric pulmonary hypertension.

Years				*p*-Value
0–2	MPA/AA ratio	Non-PHT group (*n* = 11)	1.14 (0.96–1.42)	0.0347
		PHT group (*n* = 13)	1.32 (1.21–1.48)	
	MPA/DA ratio	Non-PHT group (*n* = 11)	1.64 (1.36–1.78)	0.0089
		PHT group (*n* = 13)	2.17 (1.76–2.30)	
2–7	MPA/AA ratio	Non-PHT group (*n* = 9)	1.09 (0.84–1.31)	0.0316
		PHT group (*n* = 11)	1.25 (1.18–1.37)	
	MPA/DA ratio	Non-PHT group (*n* = 9)	1.62 (1.26–1.87)	0.0137
		PHT group (*n* = 11)	2.04 (1.78–2.36)	
7–12	MPA/AA ratio	Non-PHT group (*n* = 7)	1.21 (0.98–1.62)	0.078
		PHT group (*n* = 8)	1.34 (1.21–1.44)	
	MPA/DA ratio	Non-PHT group (*n* = 7)	1.68 (1.23–1.93)	0.0097
		PHT group (*n* = 8)	2.32 (1.89–2.45)	
12–18	MPA/AA ratio	Non-PHT group (*n* = 8)	1.23 (0.99–1.43)	0.0416
		PHT group (*n* = 9)	1.33 (1.19–1.42)	
	MPA/DA ratio	Non-PHT group (*n* = 8)	1.68 (1.32–1.91)	0.0087
		PHT group (*n* = 9)	2.24 (1.84–2.48)	
>12	MPA/AA ratio	Non-PHT group (*n* = 35)	1.16 (0.86–1.50)	0.0116
		PHT group (*n* = 41)	1.38 (1.25–1.52)	
	MPA/DA ratio	Non-PHT group (*n* = 35)	1.58 (1.36–1.67)	0.0027
		PHT group (*n* = 41)	2.2 (1.88–2.40)	

MPA: main pulmonary artery (mm), DA: descending aorta (mm), AA: ascending aorta (mm), PHT: pulmonary hypertension, Non-PHT: without pulmonary hypertension.

## Data Availability

The data that support the findings of this study are available from the corresponding author upon reasonable request. Further inquiries can be directed to the corresponding author.
